# Cardiac adaptations to 60 day head‐down‐tilt bed rest deconditioning. Findings from the AGBRESA study

**DOI:** 10.1002/ehf2.13103

**Published:** 2020-11-15

**Authors:** Fabian Hoffmann, Jérémy Rabineau, Dennis Mehrkens, Darius A. Gerlach, Stefan Moestl, Bernd W. Johannes, Enrico G. Caiani, Pierre Francois Migeotte, Jens Jordan, Jens Tank

**Affiliations:** ^1^ Institute of Aerospace Medicine German Aerospace Center Cologne Germany; ^2^ Department of Internal Medicine III University of Cologne Cologne Germany; ^3^ LPHYS Université Libre de Bruxelles Bruxelles Belgium; ^4^ Department of Electronics, Information and Biomedical Engineering Politecnico di Milano Milan Italy; ^5^ Consiglio Nazionale delle Ricerche Institute of Electronics and Information and Telecommunication Engineering Milan Italy; ^6^ Head of the Institute of Aerospace Medicine German Aerospace Center Cologne Germany

**Keywords:** Cardiac atrophy, Heart failure, Myocardial strain, Bed rest, Immobilization

## Abstract

**Aims:**

Reduced physical activity increases the risk of heart failure; however, non‐invasive methodologies detecting subclinical changes in myocardial function are not available. We hypothesized that myocardial, left ventricular, systolic strain measurements could capture subtle abnormalities in myocardial function secondary to physical inactivity.

**Methods and results:**

In the AGBRESA study, which assessed artificial gravity through centrifugation as potential countermeasure for space travel, 24 healthy persons (eight women) were submitted to 60 day strict −6° head‐down‐tilt bed rest. Participants were assigned to three groups of eight subjects: a control group, continuous artificial gravity training on a short‐arm centrifuge (30 min/day), or intermittent centrifugation (6 × 5 min/day). We assessed cardiac morphology, function, strain, and haemodynamics by cardiac magnetic resonance imaging (MRI) and echocardiography. We observed no differences between groups and, therefore, conducted a pooled analysis. Consistent with deconditioning, resting heart rate (∆8.3 ± 6.3 b.p.m., *P* < 0.0001), orthostatic heart rate responses (∆22.8 ± 19.7 b.p.m., *P* < 0.0001), and diastolic blood pressure (∆8.8 ± 6.6 mmHg, *P* < 0.0001) increased, whereas cardiac output (∆−0.56 ± 0.94 L/min, *P* = 0.0096) decreased during bed rest. Left ventricular mass index obtained by MRI did not change. Echocardiographic left ventricular, systolic, global longitudinal strain (∆1.8 ± 1.83%, *P* < 0.0001) decreased, whereas left ventricular, systolic, global MRI circumferential strain increased not significantly (∆−0.68 ± 1.85%, *P* = 0.0843). MRI values rapidly returned to baseline during recovery.

**Conclusion:**

Prolonged head‐down‐tilt bed rest provokes changes in cardiac function, particularly strain measurements, that appear functional rather than mediated through cardiac remodelling. Thus, strain measurements are of limited utility in assessing influences of physical deconditioning or exercise interventions on cardiac function.

## Background and aims

Reduced physical activity increases the risk of heart failure later in life.^1^, ^2^ Conversely, exercise interventions reverse cardiac changes associated with sedentary ageing, as determined by right heart catheterization and three‐dimensional echocardiography.^3^ To guide exercise interventions in patients, less invasive methodology is required. Echocardiographic left ventricular, systolic, global longitudinal strain predicts cardiovascular morbidity and mortality.^4^ Left ventricular, systolic, global circumferential strain analysis by magnetic resonance imaging (MRI) may further improve risk prediction.^5^ Head‐down‐tilt bed rest models cardiovascular deconditioning in weightlessness.^6^ The response resembles cardiovascular adaptation to sedentary ageing^7^ and provides a highly standardized model to assess deconditioning influences on cardiac function. We tested the hypothesis that left ventricular, systolic, myocardial strain measurements, obtained through echocardiography or MRI, could detect subclinical changes in myocardial function secondary to bed rest deconditioning. Furthermore, we determined whether artificial gravity through short‐arm centrifugation would ameliorate the response.

## Methods

This study is part of the NASA/ESA/DLR 60 day −6° head‐down‐tilt bed rest study ‘Artificial Gravity Bed Rest with European Space Agency’ (AGBRESA) conducted at the DLR: envihab. The study enrolled 24 healthy persons (23–54 years, 24.3 ± 2 kg/m^2^, eight women). We obtained written informed consent prior to study entry. The study was approved by the North Rhine Medical Association Ethics Committee and prospectively registered (DRKS00015677).

The study comprised 14 day baseline, 60 day strict −6° head‐down‐tilt bed rest, and 15 day recovery. Participants were pseudorandomly distributed to a control group, daily 6 × 5 min short‐arm centrifugation with 3 min breaks, or daily continuous 30 min short‐arm centrifugation, each with 1 Gz at the centre of mass. Participants did not exercise, were on a controlled sodium diet, and maintained a constant body weight.

We performed echocardiographic and Doppler imaging (Vivid‐IQ with M5SC‐RS sector probe, GE Healthcare, Boston, Massachusetts, USA) at baseline (supine, 6 days before bed rest) and at the end of bed rest (−6° head‐down‐tilt, 1 day before recovery) to assess biplane end‐diastolic and end‐systolic volumes; mitral annulus plane systolic excursion; left ventricular, systolic, global longitudinal peak strain by speckle tracking; transmitral filling patterns [E wave, A wave, E/A, and tissue Doppler of the lateral mitral annulus (e'lat) velocities and ratio]; and stroke volume index (derived from pulsed‐wave Doppler velocity–time integral of the left ventricular outflow tract, its diameter, and body surface area).

Cardiac MRI (3‐T Biograph, PET/MR, Siemens, Munich, Germany) was performed at baseline (5 days before bed rest), on 56th day of bed rest, and on 4th day of recovery. We recorded two‐chamber, three‐chamber, and 4‐chamber cine loops (1.6 × 1.6 × 6 mm; TE 1.43 ms, TR 39.24 ms, 25 phases) and a complete short‐axis stack (1.6 × 1.6 × 7 mm; TE 1.43 ms, TR 45.78 ms, 25 phases) with retrospective electrocardiogram gating and analysed cardiac output; left ventricular mass index; ejection fraction; left ventricular, systolic, global circumferential strain and strain rate; and time to peak (cmr42 Siemens Integration, version 5.9.3, Circle Cardiovascular Imaging Inc.) (see Appendix 1).

During passive orthostatic testing at the last day of baseline and on the last day of bed rest, we recorded resting heart rate and blood pressure.

Results are reported as mean ± standard deviation. We calculated group and time point effects using linear mixed‐effect model analysis. *P* < 0.05 indicated statistical significance. The data supporting the reported results are available from the corresponding author upon reasonable request.

## Results

**Figure 1:**
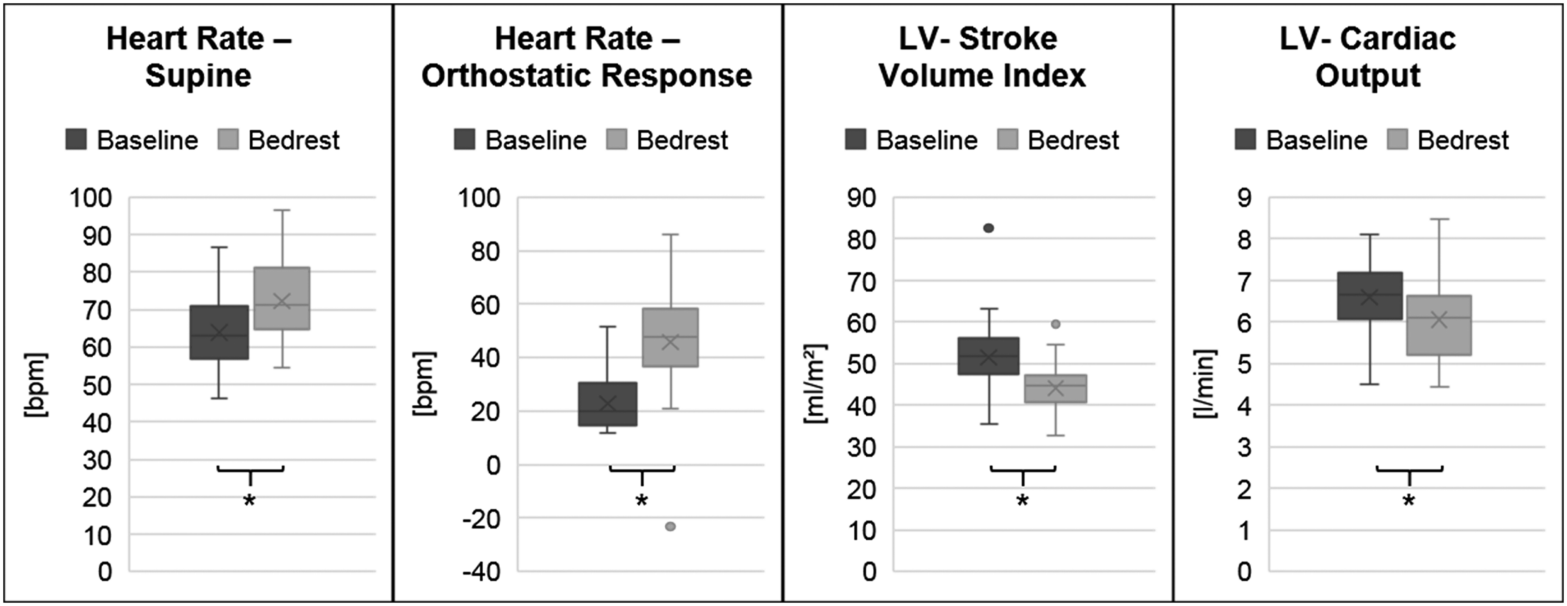
Cardiac deconditioning. Supine and upright heart rate, left ventricular (LV) stroke volume index, and cardiac output at baseline and after 60 day bed rest. *P < 0.05.

Because baseline characteristics and cardiac responses did not differ between groups (Appendix 1), we conducted a pooled analysis in all 24 participants (*Table* *1*). Compared with baseline, supine heart rate increased 8.3 ± 6.3 b.p.m. (*P* < 0.0001), systolic blood pressure did not change, and diastolic blood pressure increased 8.8 ± 6.6 mmHg (*P* < 0.0001) at the end of bed rest. On Day 4 of recovery, blood pressure had returned to baseline, while resting heart rate remained elevated by 5.6 ± 8.4 b.p.m. (*P* < 0.001). With standing, heart rate increased 22.8 ± 10.5 b.p.m. at baseline and 45.6 ± 21.4 b.p.m. following bed rest (*P* < 0.0001; *Figure* *1*).

**Table 1:** Cohort analysis

		Baseline	Bed rest	Recovery	*P*
Heart rate	(b.p.m.)	64 ± 9.6	72.3 ± 10.6	69.6 ± 10.5	<0.0001
Systolic blood pressure	(mmHg)	125 ± 11.1	124.3 ± 8.9	122.7 ± 70.6	0.561
Diastolic blood pressure	(mmHg)	69.6 ± 7.3	78.5 ± 6.9	70.3 ± 6.3	<0.0001
∆Upright–supine heart rate	(b.p.m.)	22.8 ± 10.5		45.6 ± 21.4	<0.0001
Cardiac output^c^	(L/min)	6.6 ± 0.9	6 ± 1	6.8 ± 1.2	0.015
Ejection fraction^b^ ^,^ ^c^	(%)	68.3 ± 3.9	66.4 ± 4.8	63.9 ± 4.7	0.005
LV mass index^c^	(g/m^2^)	66.6 ± 11.3	64.5 ± 11.7	65.8 ± 9.8	0.792
LV stroke volume index^d^	(mL/^2^)	51.5 ± 10	44.1 ± 6.3		0.001
LV EDV^d^	(mL)	100.1 ± 28.2	79.7 ± 17.6		<0.0001
MAPSE^d^	(mm)	18.5 ± 2.7	16.6 ± 3.1		0.013
Global longitudinal PS^d^	(%)	−19.9 ± 2.1	−18.1 ± 2.1		<0.0001
Global circumferential PS^a^ ^,^ ^c^	(%)	−18.6 ± 1.7	−19.1 ± 1.6	−18.1 ± 1.7	0.049
Global circumferential sSR^c^	(1/s)	−0.97 ± 0.1	−1.14 ± 0.18	−1 ± 0.11	<0.0001
Global circumferential t2p^c^	(ms)	315 ± 35.1	285.9 ± 28.6	306.9 ± 25.2	<0.0001
E‐wave velocity^d^	(cm/s)	79.4 ± 14.1	65.3 ± 12.5		<0.0001
A‐wave velocity^d^	(cm/s)	52.7 ± 13	53.3 ± 12.1		0.796
E/A ratio^d^		1.58 ± 0.39	1.25 ± 0.24		0.015
e'lateral^d^	(cm/s)	15.5 ± 2.9	12.3 ± 2.7		<0.0001
E/e'lateral ratio^d^		5.25 ± 1.17	5.68 ± 1.66		0.0889

LV, left ventricular; LV EDV, left ventricular end‐diastolic volume; MAPSE, mitral annulus plane systolic excursion; PS, peak strain; sSR, systolic strain rate; t2p, time to systolic peak strain.

Absolute mean values ± standard deviation of the whole cohort for all three time points (baseline, bed rest, and recovery). *P*‐values for linear mixed‐effect model analysis. *P* < 0.05 indicates significance. All strain measurements refer to the left ventricle in systole. All strain values refer to the left ventricle in systole.

^a^ In pairwise comparison of baseline vs. bed rest and baseline vs. recovery, values do not differ significantly.

^b^ In pairwise comparison of baseline vs. recovery, results differ significantly (*P* = 0.005).

^c^ Parameters obtained by cardiac magnetic resonance imaging.

^d^ Parameters obtained by echocardiography.

**Figure 2:**
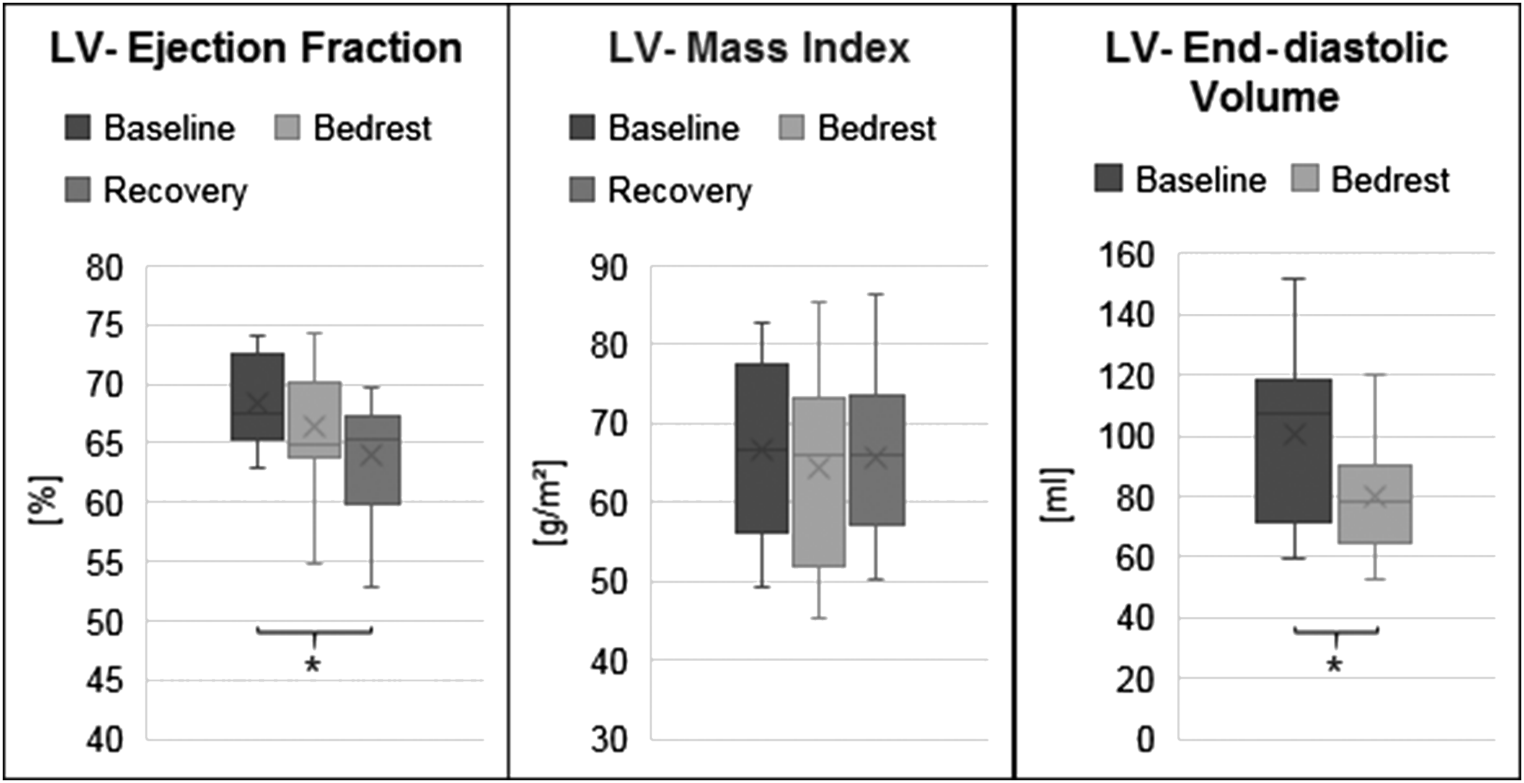
Left ventricular (LV) function and morphology. LV ejection fraction, LV mass index derived from cardiac magnetic resonance imaging at baseline, after 60 day bed rest, and recovery. LV end‐diastolic volume by echocardiography at baseline and after 60 day bed rest. *P < 0.05.

Following bed rest, cardiac output and left ventricular stroke volume index had decreased 8.2% (−0.54 ± 0.94 L/min, *P* = 0.0096) and 14.4% (−7.4 ± 8.3 mL/m^2^, *P* = 0.0168), respectively. Left ventricular end‐diastolic volume determined by echocardiography decreased 20.3 ± 15.4% (*P* = 0.0001) together with ejection fraction (6.4 ± 5.1%). Left ventricular mass index did not change (*Figure* *2*). Left ventricular mass index by MRI, which was significantly greater in men compared with women (*P* = 0.0001), did not change in men (baseline: 70.4 ± 10.7; recovery: 68.7 ± 8.6 g/m^2^, *P* = 0.69) or in women (baseline: 59 ± 8.6; recovery: 59.8 ± 9.9 g/m^2^, *P* = 0.968). Mitral annulus plane systolic excursion and global longitudinal peak strain were reduced following bed rest (*Table* *1*).

**Figure 3:**
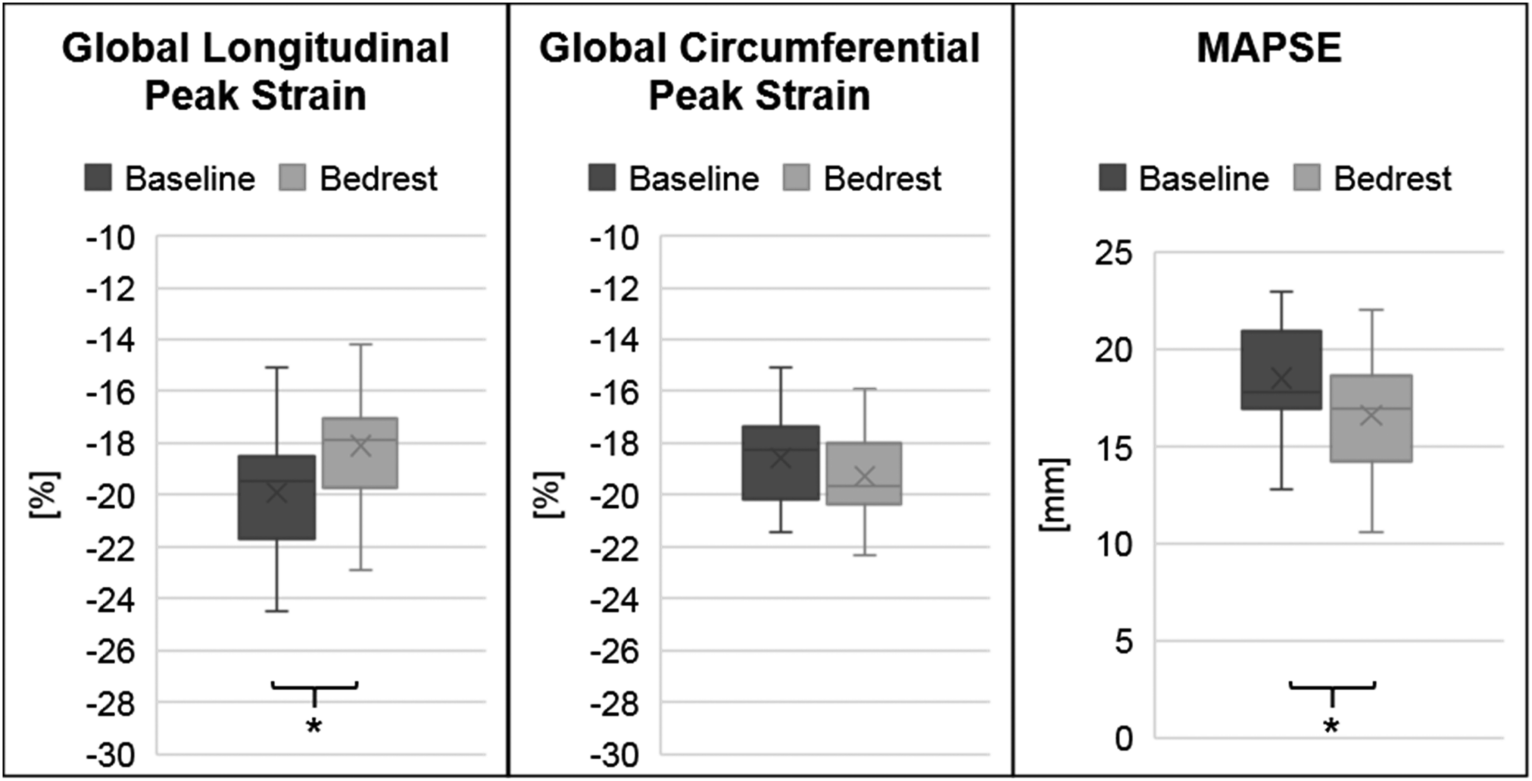
Cardiac strain. Cardiac strain measurements at baseline and after 60 day bed rest (56 days for circumferential strain). *P < 0.05. MAPSE, mitral annulus plane systolic excursion.

**Figure 4:**
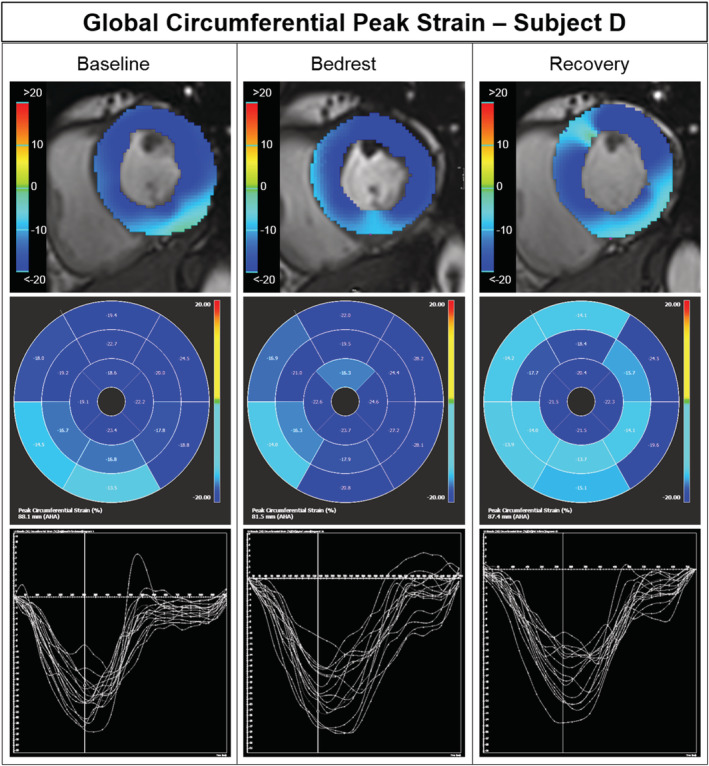
Global circumferential peak strain. Circumferential peak strain measurements at baseline, end of bed rest, and end of recovery in a representative study participant. Upper panel: end‐systolic cross‐sectional short‐axis cardiac magnetic resonance imaging images at the level just above the papillary muscles with circumferential strain overlay. Middle panel: Bull's eye view of the 16 American Heart Association (AHA) myocardial segments model with circumferential peak strain values and colour coding, where deeper blue resembles higher strain values. Lower panel: Circumferential peak strain time course over one heartbeat for the 16 AHA myocardial segments model.

Left ventricular, systolic global circumferential peak strain by cardiac MRI did not change significantly with bed rest (*Figure* *3*). However, following 4 day recovery, global circumferential peak strain tended to decrease compared with bed rest (*P* = 0.05; *Figure* *4*). Circumferential contraction expressed as systolic strain rate and time to peak was significantly augmented at Day 56 of bed rest compared with baseline with increases in strain rate and shortened time to peak. While peak values for transmitral A wave did not change with bed rest, E was reduced such that the E/A ratio decreased. We observed a similar pattern for e'lat, whereas E/e'lat remained unchanged.

Artificial gravity through intermittent or continuous centrifugation did not abolish cardiovascular adaptations to head‐down‐tilt bed rest (Appendix 1).

## Discussion

Sixty days of strict head‐down‐tilt bed rest elicited cardiovascular deconditioning indicated by increases in resting and upright heart rate with reductions in left ventricular end‐diastolic volume, cardiac output, and stroke volume. Yet bed rest did not lead to clinical apparent heart failure. Previous studies showed worsened cardiopulmonary fitness and orthostatic tolerance.^8^ Yet we did not observe sustained reductions in left ventricular function assessed by systolic strain analysis in line with shorter duration bed rest studies.^9^ Finally, myocardial mass did not change significantly, suggesting that cardiac atrophy is not a general feature during physical deconditioning and cannot be seen as risk factor for developing chronic heart failure. While we cannot exclude modest improvements in cardiovascular deconditioning, artificial gravity failed to abolish the response.

Strain can be affected by intrinsic myocardial properties, cardiac loading conditions, and sympathetic drive.^10^ We and others observed reductions in left ventricular end‐diastolic volume with predominant long‐axis diameter shortening following bed rest deconditioning.^11^ The phenomenon may result from plasma volume reductions during bed rest.^12^, ^13^ Plasma volume reductions are at least in part explained by cephalad volume shifts promoting natriuretic peptide release through atrial stretch.^14^, ^15^, ^16^, ^17^ The left ventricle seems less compliant with a smaller stroke volume independent of the volume loss.^14^ The asymmetric change in left ventricular shape likely explains differential global circumferential and longitudinal strain responses.^10^ Normalization of strain and left ventricular volumes within days of recovery is consistent with loading‐dependent functional changes rather than cardiac remodelling that might lead to persistent cardiac dysfunction. Left ventricular diastolic filling, which is also preload dependent, changed as well.^18^, ^19^ Similar volume alterations have been reported during 5 and 35 days of bed rest.^13^, ^20^ Altered loading conditions may also explain the significant albeit small reduction in left ventricular ejection fraction upon recovery.

Cardiac function measurements could be confounded by sympathetic activation, which is an expected physiological response to plasma volume reductions. Indeed, increases in resting heart rate and diastolic blood pressure, which we observed at the end of bed rest similar to others,^13^ often occur in conditions associated with increased sympathetic drive.^21^, ^22^ Previous findings in bed rest studies support the idea that sympathetic activity is, indeed, increased.^23^, ^24^, ^25^ Furthermore, after 21 day bed rest, plasma norepinephrine increased more with orthostasis compared with baseline.^26^ We speculate that sympathetic activation may have increased circumferential strain with bed rest.

The main limitation of our study is the relatively small sample size limiting statistical power and detailed subgroup analyses. Yet rigorous standardization including controlled sodium intake and caloric adjustment to maintain body weight made it possible observing small but relevant physiological changes in cardiovascular function. Furthermore, participants were relatively young with low heart failure risk. Finally, longer periods of limited physical activity may be required to alter intrinsic myocardial properties and to promote interstitial fibrosis.

We conclude that 60 days of −6° head‐down‐tilt bed rest provoke changes in cardiac function that appear functional rather than mediated through cardiac remodelling. Additional risks such as older age or concomitant cardiovascular disease may be required to express cardiac dysfunction and consecutive chronic heart failure. Because −6° head‐down‐tilt bed rest is a model for weightless conditions, our findings are reassuring for human space travel. While in weightlessness, cardiopulmonary fitness and orthostatic tolerance will deteriorate in the absence of sufficient countermeasures, overt cardiac disease appears unlikely. Furthermore, our findings might have implications for patients undergoing forced bed rest in, for example, intensive care settings. Finally, our study suggests that strain measurements, as preload‐dependent analysis, may be of limited utility in prospectively guiding exercise interventions in the prevention of heart failure. While deconditioning elicits plasma volume reductions and sympathetic activation, physical exercise, particularly endurance training, elicits the opposite response.^27^, ^28^ Thus, intrinsic changes in myocardial functional properties cannot be discerned.

## Conflict of interest

None.

## Funding

This work was supported by NASA, ESA, the Belgian Federal Scientific Policy Office (PRODEX PEA 4000110826), and programmatic funding of the German Aerospace Center (DLR). F.H. received funding from the German Aerospace Center (DLR) and the German Federal Ministry of Economy and Technology (BMWi; 50WB1816). J.R. was supported by the Fonds de la Recherche Scientifique (Mandat Aspirant F.R.S.–FNRS FC 29801).

## Appendix

**Table:**

		Baseline				
		Total	Control	Continuous AG	Intermittent AG	*P*
Weight	(kg)	74 ± 10.1	79.5 ± 12.7	71.3 ± 9.9	71.3 ± 4.8	0.1709
Height	(cm)	174.4 ± 8.7	176.9 ± 7.3	172.1 ± 8.1	174.1 ± 10.7	0.566
Body surface area	(m^2^)	1.89 ± 0.169	1.96 ± 0.19	1.84 ± 0.17	1.85 ± 0.14	0.2874
Age	(years)	33.3 ± 9.3	33.8 ± 8.2	31.4 ± 9.9	34.6 ± 10.6	0.7855
Heart rate	(b.p.m.)	64 ± 9.6	63.8 ± 7	63.4 ± 13.2	63.4 ± 9	0.9752
Systolic blood pressure	(mmHg)	125 ± 11.1	125.2 ± 8.2	127 ± 14.9	122.9 ± 10.2	0.78
Diastolic blood pressure	(mmHg)	69.6 ± 7.3	71 ± 8.2	70.3 ± 6.4	67.5 ± 7.6	0.6187
Cardiac output	(L/min)	6.59 ± 0.89	6.75 ± 0.85	6.57 ± 0.89	6.44 ± 1	0.7935
Ejection fraction	(%)	68.3 ± 3.9	66.6 ± 3.4	70.5 ± 4	67.8 ± 3.5	0.11
LV mass index	(g/m^2^)	66.6 ± 11.3	64.4 ± 13	69.7 ± 11.6	65.7 ± 9.9	0.643
LV stroke volume index	(mL/^2^)	51.5 ± 10	50.5 ± 14.3	50.8 ± 9.8	53.2 ± 4.7	0.8566
LV EDV	(mL)	100.1 ± 28.2	109.2 ± 34.9	98.5 ± 29.3	92.6 ± 19.5	0.5095
MAPSE	(mm)	18.5 ± 2.7	17.5 ± 3	18.3 ± 2.8	19.7 ± 2.1	0.276
Global longitudinal PS	(%)	−19.9 ± 2.1	−19.7 ± 2.2	−19.8 ± 1.7	−20.2 ± 2.6	0.8966
Global circumferential PS	(%)	−18.6 ± 1.7	−18.6 ± 1.6	−18.3 ± 2	−18.8 ± 1.6	0.8181
Global circumferential sSR	(1/s)	−0.97 ± 0.1	−0.98 ± 0.11	−0.97 ± 0.12	−0.99 ± 0.08	0.958
Global circumferential t2p	(ms)	315 ± 35.1	312.3 ± 34.7	323.3 ± 47.1	303 ± 19.6	0.5296
E‐wave velocity	(cm/s)	79.4 ± 14.8	79.6 ± 17.1	80.9 ± 9.6	77.6 ± 16.4	0.9053
A‐wave velocity	(cm/s)	52.7 ± 13	56.5 ± 15.1	53 ± 15.7	48.6 ± 6.2	0.4993
E to A ratio		1.58 ± 0.39	1.5 ± 0.45	1.62 ± 0.38	1.61 ± 0.39	0.8078
e'lateral	(cm/s)	15.5 ± 2.9	14.8 ± 2.5	16.3 ± 3.8	15.1 ± 2.3	0.561
E to e'lateral ratio		5.25 ± 1.17	5.47 ± 1.48	5.17 ± 1.17	5.17 ± 0.97	0.8646

LV, left ventricular; LV EDV, left ventricular end‐diastolic volume; MAPSE, mitral annulus plane systolic excursion; PS, peak strain; sSR, systolic strain rate; t2p, time to systolic peak strain.

Baseline characteristics: Absolute mean values ± standard deviation of the whole cohort and three subgroups [control, continuous artificial gravity (AG) and intermittent AG] at baseline. *P*‐values for linear mixed‐effect model analysis. *P* < 0.05 indicates significance. All strain measurements refer to the left ventricle in systole.

**Table:**

		∆Bed rest—Baseline				∆Recovery—Baseline			
		Control	Continuous AG	Intermittent AG	*P*	Control	Continuous AG	Intermittent AG	*P*
Heart rate	(b.p.m.)	8.2 ± 7.6	9 ± 4.6	7.6 ± 7.1	0.9021	7.7 ± 5.3	9.4 ± 8	0.2 ± 9.2	0.0616
Systolic blood pressure	(mmHg)	3.4 ± 7.2	−3.1 ± 13.5	−2.5 ± 6.7	0.3459	−1.5 ± 7.6	0.6 ± 7.9	6 ± 8.9	0.3729
Diastolic blood pressure	(mmHg)	9.1 ± 6.8	8.6 ± 5.9	8.8 ± 8.0	0.8677	−1 ± 4.7	2.5 ± 2.4	0.6 ± 6.8	0.3882
Cardiac output	(L/min)	−0.51 ± 0.81	−0.41 ± 1.1	−0.76 ± 0.99	0.7657	0.07 ± 0.77	0.54 ± 1.13	0.18 ± 1.02	0.3729
Ejection fraction	(%)	−2.06 ± 5.19	−1.69 ± 5.06	−2.08 ± 5.1	0.985	−4.36 ± 3.09	−4.3 ± 3.8	−4.43 ± 3.93	0.998
LV mass index	(g/m^2^)	−0.58 ± 6.43	−5 ± 3.97	−0.84 ± 5.82	0.222	−1.95 ± 5.21	−2.59 ± 6.05	2.04 ± 7.56	0.308
LV stroke volume index	(mL/^2^)	−7.3 ± 12.3	−8.3 ± 6.2	−6.6 ± 6	0.926				
LV EDV	(mL)	−26.8 ± 29.2	−16.1 ± 15	−18.1 ± 10	0.3181				
MAPSE	(mm)	−0.69 ± 3.06	−3.28 ± 3	−1.72 ± 4.12	0.335				
Global longitudinal PS	(%)	−2.03 ± 1.54	−2.36 ± 1.28	−1.01 ± 2.42	0.3181				
Global circumferential PS	(%)	0.15 ± 2.21	−1.34 ± 1.77	−0.86 ± 1.38	0.2676	1.7 ± 1.59	−0.39 ± 1.53	−0.05 ± 1.91	0.054
Global circumferential sSR	(1/s)	−0.16 ± 0.16	−0.25 ± 0.2	−0.18 ± 0.12	0.5263	0.01 ± 0.14	−0.08 ± 0.15	−0.03 ± 0.15	0.4863
Global circumferential t2p	(ms)	−23.2 ± 33.7	−38.8 ± 40	24.3 ± 31.1	0.6181	0.0 ± 21.6	−23.1 ± 40.8	−3.1 ± 37.6	0.3897
E‐wave velocity	(cm/s)	−2 ± 18	−11.3 ± 13.7	−11 ± 10.2	0.3867				
A‐wave velocity	(cm/s)	−7.5 ± 12.3	3.5 ± 18.6	5.9 ± 14.3	0.1992				
E to A ratio		−0.26 ± 0.34	−0.34 ± 0.54	−0.36 ± 0.42	0.883				
e'lateral	(cm/s)	−4.3 ± 2.3	−3.1 ± 2.3	−2.4 ± 1.8	0.8168				
E to e'lateral ratio		0.33 ± 2.65	0.25 ± 1.33	0.42 ± 2.10	0.741				

LV, left ventricular; LV EDV, left ventricular end‐diastolic volume; MAPSE, mitral annulus plane systolic excursion; PS, peak strain; sSR, systolic strain rate; t2p, time to systolic peak strain.

Intergroup comparison: Differences of bed rest—baseline and recovery—baseline ± standard deviation of the whole cohort and three subgroups [control, continuous artificial gravity (AG), and intermittent AG]. All strain measurements refer to the left ventricle in systole. *P* < 0.05 indicates significance.

## Cardiac magnetic resonance imaging acquisition parameters

Two‐chamber, three‐chamber, and four‐chamber views and right ventricular long‐axis view—cine

**Table:**

CINE_3CV_4CV_RV_2CV	
TA: 3.2 s PM: REF voxel size: 1.6 × 1.6 × 6.0 mm PAT: 2 Rel. SNR: 1.00: tfi	
Properties	
Prio recon	Off
Load images to viewer	On
Inline movie	On
Auto store images	On
Load images to stamp segments	On
Load images to graphic segments	On
Auto open inline display	Off
Auto close inline display	Off
Start measurement without further preparation	Off
Wait for user to start	Off
Start measurements	Single measurement
Routine	
Slice group	1
AutoAlign	—
Phase oversampling	50%
FoV read	340 mm
FoV phase	83.7%
Slice thickness	6.0 mm
TR	39.24 ms
TE	1.43 ms
Averages	1
Concatenations	1
Filter	Distortion corr. (2D)
	Prescan normalize
	Image filter
Coil elements	BP1, 2; SP1–3
Slices	1
Dist. factor	20%
Position	L4.2 A1.0 H24.6 mm
Orientation	T > C32.0 > S‐12.2
Phase enc. dir.	A >> P
Contrast—Common	
TR	39.24 ms
TE	1.43 ms
Magn. preparation	None
Flip angle	40°
Fat suppr.	None
Wrap‐up magn.	Restore
Contrast—Dynamic	
Averages	1
Averaging mode	Short term
Reconstruction	Magnitude
Measurements	1
Multiple series	Each slice
Resolution—Common	
FoV read	340 mm
FoV phase	83.70%
Slice thickness	6.0 mm
Base resolution	208
Phase resolution	80%
Phase partial Fourier	Off
Trajectory	Cartesian
View sharing	Off
Interpolation	Off
Resolution—iPAT	
PAT mode	GRAPPA
Accel. factor PE	2
Ref. lines PE	24
Matrix coil mode	Auto (triple)
Reference scan mode	Integrated
Resolution—Filter image	
Image filter	On
! Intensity	Medium
Edge enhancement	1
Smoothing	3
Unfiltered images	Off
Distortion corr.	On
Mode	2D
Unfiltered images	Off
Prescan normalize	On
Unfiltered images	Off
Normalize	Off
B1 filter	Off
Resolution—Filter raw data	
Raw filter	Off
Elliptical filter	Off
POCS	Off
Geometry—Common	
Slice group 1	
FoV read	340 mm
FoV phase	83.7%
Slice thickness	6.0 mm
TR	39.24 ms
Multi‐slice mode	Sequential
Series	Descending
Concatenations	1
Slices 1	
Dist. factor 20%	
Position L4.2 A1.0 H24.6 mm	
Orientation T > C32.0 > S‐12.2	
Phase enc. dir. A >> P	
Geometry—AutoAlign	
Slice group	1
AutoAlign	—
Position	L4.2 A1.0 H24.6 mm
Orientation	T > C32.0 > S‐12.2
Phase enc. dir.	A >> P
Initial position	Isocentre
L	0.0 mm
P	0.0 mm
H	0.0 mm
Initial rotation	0.00°
Initial orientation	Transversal
Geometry—Saturation	
Fat suppr.	None
Wrap‐up magn.	Restore
Special sat.	None
Geometry—Navigator	
Geometry—Tim planning suite	
Set‐n‐Go protocol	Off
Table position	H
Table position	0 mm
Inline composing	Off
System—Miscellaneous	
Positioning mode REF	
Table position H	
Table position 0 mm	
MSMA S‐C‐T	
Sagittal R >> L	
Coronal A >> P	
Transversal F >> H	
Coil combine mode sum of squares	
Save uncombined off	
Matrix coil mode auto (triple)	
AutoAlign—	
Coil select mode off—AutoCoilSelect	
System—Adjustments	
B0 Shim mode	Cardiac
B1 Shim mode	TrueForm
Adjust with body coil	Off
Confirm freq. adjustment	Off
Assume dominant fat	Off
Assume silicone	Off
Adjustment tolerance	Auto
System—Adjust volume	
Position	L4.2 A1.0 H24.6 mm
Orientation	T > C32.0 > S‐12.2
Rotation	7.56°
A >> P	285 mm
R >> L	340 mm
F >> H	6 mm
Reset	Off
System—Tx/Rx	
Frequency 1H	123.197081 MHz
Correction factor	1
Gain	High
Img. scale cor.	1.000
Reset	Off
? Ref. amplitude 1H	0.000 V
Physio—Signal1	
1st signal/mode	ECG/retro
Average cycle	290 ± 23 ms
Average cycle	No signal ms
Calculated phases	25
TR	39.24 ms
Concatenations	1
Segments	12
Arrhythmia detection	None
Physio—Cardiac	
Tagging	None
Magn. preparation	None
Fat suppr.	None
Dark blood	Off
FoV read	340 mm
FoV phase	83.70%
Phase resolution	80%
Cine	On
Physio—Cardiac	
Trajectory	Cartesian
View sharing	Off
Dummy heartbeats	1
Physio—PACE	
Resp. control	Breath‐hold
Concatenations	1
Inline—Common	
Subtract	Off
Measurements	1
StdDev	Off
Save original images	On
Inline—Cardiac	
Inline evaluation	Ventricular function
Magn. preparation	None
Contrasts	1
TE	1.43 ms
TR	39.24 ms
Save original images	On
Inline—MIP	
MIP‐Sag	Off
MIP‐Cor	Off
MIP‐Tra	Off
MIP‐Time	Off
Save original images	On
Inline—Composing	
Inline composing	Off
Distortion corr.	On
Mode	2D
Unfiltered images	Off
Sequence—Part 1	
Introduction	Off
Dimension	2D
Reordering	Linear
Asymmetric echo	Weak
Contrasts	1
Optimization	Min. TE TR
Multi‐slice mode	Sequential
Echo spacing	3.3 ms
Sequence type	Trufi
Bandwidth	962 Hz/Px
Sequence—Part 2	
Define	Segments
Segments	12
Trufi delta freq.	0 Hz
RF pulse type	Normal
Gradient mode	Fast
Excitation	Slice‐sel.
Flip angle mode	Constant
Cine	On
Sequence—Assistant	
Mode	Min flip angle
Min flip angle	45°
Allowed delay	5 s

Left ventricular short‐axis stack—cine

**Table:**

CINE_segmented_SAX*	
TA: 2.0 s PM: REF voxel size: 1.6 × 1.6 × 7.0 mm PAT: 3 Rel. SNR: 1.00: tfi	
Properties	
Prio recon	Off
Load images to viewer	On
Inline movie	On
Auto store images	On
Load images to stamp segments	On
Load images to graphic segments	On
Auto open inline display	Off
Auto close inline display	Off
Start measurement without further preparation	Off
Wait for user to start	Off
Start measurements	Single measurement
Routine	
Slice group	1
AutoAlign	—
Phase oversampling	50%
FoV read	340 mm
FoV phase	80.8%
Slice thickness	7.0 mm
TR	45.78 ms
TE	1.43 ms
Averages	1
Concatenations	1
Filter	Distortion corr. (2D)
	Prescan normalize
	Image filter
Coil elements	BP1, 2; SP1–3
Slices	1
Dist. factor	20%
Position	L4.2 A1.0 H24.6 mm
Orientation	T > C32.0 > S‐12.2
Phase enc. dir	A >> P
Contrast—Common	
TR	45.78 ms
TE	1.43 ms
Magn. preparation	None
Flip angle	40°
Fat suppr.	None
Wrap‐up magn.	Restore
Contrast—Dynamic	
Averages	1
Averaging mode	Short term
Reconstruction	Magnitude
Measurements	1
Multiple series	Each slice
Resolution—Common	
FoV read	340 mm
FoV phase	80.80%
Slice thickness	7.0 mm
Base resolution	208
Phase resolution	70%
Phase partial Fourier	Off
Trajectory	Cartesian
View sharing	Off
Interpolation	Off
Resolution—iPAT	
PAT mode	GRAPPA
Accel. factor PE	3
Ref. lines PE	24
Matrix coil mode	Auto (triple)
Reference scan mode	Integrated
Resolution—Filter image	
Image filter	On
! Intensity	Medium
Edge enhancement	1
Smoothing	3
Unfiltered images	Off
Distortion corr.	On
Mode	2D
Unfiltered images	Off
Prescan Normalize	On
Unfiltered images	Off
Normalize	Off
B1 filter	Off
Resolution—Filter raw data	
Raw filter	Off
Elliptical filter	Off
POCS	Off
Geometry—Common	
Slice group 1	
FoV read	340 mm
FoV phase	80.8%
Slice thickness	7.0 mm
TR	45.78 ms
Multi‐slice mode	Sequential
Series base	To apex
Concatenations	1
Slices	1
Dist. factor	20%
Position	L4.2 A1.0 H24.6 mm
Orientation	T > C32.0 > S‐12.2
Phase enc. dir.	A >> P
Geometry—AutoAlign	
Slice group	1
AutoAlign	—
Position	L4.2 A1.0 H24.6 mm
Orientation	T > C32.0 > S‐12.2
Phase enc. dir.	A >> P
Initial position	Isocentre
L	0.0 mm
P	0.0 mm
H	0.0 mm
Initial rotation	0.00°
Initial orientation	Transversal
Geometry—Saturation	
Fat suppr.	None
Wrap‐up magn.	Restore
Special sat.	None
Geometry—Navigator	
Geometry—Tim planning suite	
Set‐n‐Go protocol	Off
Table position	H
Table position	0 mm
Inline composing	Off
System—Miscellaneous	
Positioning mode	REF
Table position	H
Table position	0 mm
MSMA	S‐C‐T
Sagittal	R >> L
Coronal	A >> P
Transversal	F >> H
Coil combine mode	Sum of squares
Save uncombined	Off
Matrix coil mode	Auto (triple)
AutoAlign	—
Coil select mode	Off—AutoCoilSelect
System—Adjustments	
B0 Shim mode	Cardiac
B1 Shim mode	TrueForm
Adjust with body coil	Off
Confirm freq. adjustment	Off
Assume dominant fat	Off
Assume silicone	Off
Adjustment tolerance	Auto
System—Adjust volume	
Position	L4.2 A1.0 H24.6 mm
Orientation	T > C32.0 > S‐12.2
Rotation	7.56°
A >> P	275 mm
R >> L	340 mm
F >> H	7 mm
Reset	Off
System—Tx/Rx	
Frequency 1H	123.197081 MHz
Correction factor	1
Gain	High
Img. scale cor.	1.000
Reset	Off
? Ref. amplitude 1H	0.000 V
Physio—Signal1	
1st signal/mode	ECG/retro
Average cycle	290 ± 23 ms
Average cycle	No signal ms
Calculated phases	25
TR	45.78 ms
Concatenations	1
Segments	14
Arrhythmia detection	None
Physio—Cardiac	
Tagging	None
Magn. preparation	None
Fat suppr.	None
Dark blood	Off
FoV read	340 mm
FoV phase	80.80%
Phase resolution	70%
Cine	On
Physio—Cardiac	
Trajectory	Cartesian
View sharing	Off
Dummy heartbeats	1
Physio—PACE	
Resp. control	Breath‐hold
Concatenations	1
Inline—Common	
Subtract	Off
Measurements	1
StdDev	Off
Save original images	On
Inline—Cardiac	
Inline evaluation	Ventricular function
Magn. preparation	None
Contrasts	1
TE	1.43 ms
TR	45.78 ms
Save original images	On
Inline—MIP	
MIP‐Sag	Off
MIP‐Cor	Off
MIP‐Tra	Off
MIP‐Time	Off
Save original images	On
Inline—Composing	
Inline composing	Off
Distortion corr.	On
Mode	2D
Unfiltered images	Off
Sequence—Part 1	
Introduction	Off
Dimension	2D
Reordering	Linear
Asymmetric echo	Weak
Contrasts	1
Optimization	Min. TE TR
Multi‐slice mode	Sequential
Echo spacing	3.3 ms
Sequence type	Trufi
Bandwidth	962 Hz/Px
Sequence—Part 2	
Define	Segments
Segments	14
Trufi delta freq.	0 Hz
RF pulse type	Normal
Gradient mode	Fast
Excitation	Slice‐sel.
Flip angle mode	Constant
Cine	On
Sequence—Assistant	
Mode	Min flip angle
Min flip angle	45°
Allowed delay	5 s
